# A machine learning aided interpretable model for rupture strength prediction in Fe-based martensitic and austenitic alloys

**DOI:** 10.1038/s41598-021-83694-z

**Published:** 2021-03-09

**Authors:** Osman Mamun, Madison Wenzlick, Jeffrey Hawk, Ram Devanathan

**Affiliations:** 1 Energy and Environment Directorate, Pacific Northwest National Laboratory, Richland, USA; 2 Materials Performance Division, National Energy Technology Laboratory, 1450 Queen Avenue SW, Albany, OR 97321 USA; 3 Leidos Research Support Team, 1450 Queen Avenue SW, Albany, OR 97321 USA

**Keywords:** Computational methods, Metals and alloys, Mechanical properties

## Abstract

The class of 9–12% Cr ferritic-martensitic alloys (FMA) and austenitic stainless steels have received considerable attention due to their numerous applications in high temperature power generation industries. To design high strength steels with prolonged service life requires a thorough understanding of the long-term properties, e.g., creep rupture strength, rupture life, etc., as a function of the chemical composition and processing parameters that govern the microstructural characteristics. In this article, the creep rupture strength of both 9–12% Cr FMA and austenitic stainless steel has been parameterized using curated experimental datasets with a gradient boosting machine. The trained model has been cross validated against unseen test data and achieved high predictive performance in terms of correlation coefficient ($$R^{2} > 0.98 $$ for 9–12% Cr FMA and $$R^{2} > 0.95 $$ for austenitic stainless steel) thus bypassing the need for additional comprehensive tensile test campaigns or physical theoretical calculations. Furthermore, the feature importance has been computed using the Shapley value analysis to understand the complex interplay of different features.

## Introduction

Austenitic stainless steels are used ubiquitously in power plants, mainly because of their excellent corrosion resistance properties, operating at high temperatures $( > 650\;^\circ {\text{C}})$ and pressures (> 50 MPa)^1–4^. However, their inability to keep their crystal structure intact during cooling renders them extremely difficult to utilize for a long service life^5,6^. In contrast, ferritic-martensitic steels offer good resistance to creep within their creep stability range, but they are prone to oxidation in steam and corrosive degradation in harsh fireside environments^7–9^. For a fossil energy power plant, it is essential to have as high an operating temperature as possible with prolonged alloy rupture life and rupture strength to ensure high thermodynamic efficiency, less carbon emission, and cost-effective operations. In order to push the temperature and pressure envelope of a modern fossil energy power plant to higher levels, a systematic investigation of the chemical and mechanical factors affecting the rupture life and rupture strength is desired to (1) have more control over the chemical composition and processing parameters that will yield the desired physical and mechanical properties, and (2) confidently assess the performance of a newly developed alloy with limited testing.

Given the importance of ultra-high strength (UHS) materials, such as austenitic stainless steels and 9–12% Cr FMA, it is essential to understand the relation between physical and mechanical processing parameters, along with the chemical compositions of the various constituent elements, and the yield strength of ferrous materials. In order to obtain fundamental understanding about the functional interplay of these variables, several models have been developed, e.g., 1. Microstructural evolution and the correlations between composition/processing and microstructure to characterize the microstructure based on the composition/processing parameters, 2. Microstructure/property relationship to characterize the property based on the microstructure etc. These models led to the systematic development of high strength ferrous alloy materials through the experimental trial and error approach; however, due to the inefficient and limiting capacity of the experimental trial and error, the development of novel materials with superior property has been very time and cost prohibitive^10–12^.

To boost the discovery of high strength ferrous materials, experimental trial and error combined with physics-based constitutive equations and/or computational CALPHAD^13^ evaluations have become the main approaches for developing predictive models for rupture life or rupture strength^14^. As a result, the concerted use of density functional theory^15,16^, Monte Carlo simulations^17^, molecular dynamics approach^18^, and phase-field models^19–21^ led to the rapid development of advanced simulation techniques to enable novel materials discovery. Recently, data science based approaches, such as machine learning (ML), are quickly emerging as powerful tools for building accurate and reliable predictive models to shorten the development time compared to traditional experimental and computational approaches^14,22^. Machine learning enables a computational approach to finding latent rules in the data so that they can be exploited for making future predictions without any active human intervention (in theory)^23^. With the advent of the Materials Genome Initiative, highly sophisticated database management system (DBMS), and unprecedented improvement in machine learning algorithms and computational power, machine learning has enabled development of highly accurate and fast predictive models that are accelerating the identification and subsequent deployment of superior materials for a variety of applications^24,25^. In a recent assessment of the literature, a data science based approach has been found to be more accurate than just a physics-based one, or one using thermodynamics-based models, for the prediction of rupture life or rupture strength^26–29^. Gaussian Process regression with Matérn kernel has been successfully utilized to predict the creep rupture life with 56% overall prediction performance by synergistically exploiting the experimental findings with the state-of-the-art computational machine learning methods^26^. Another article showed that by incorporating thermodynamics data generated from the computational thermodynamics study into the machine learning model, highly accurate models can be obtained for creep prediction in ferrous materials^27^. In a recent article, Jiang et al. showed that a machine learning model can be successfully employed to accurately predict the tensile strength in pearlitic steel wires^28^. However, the assessment also revealed a number of weaknesses in the existing ML models infrastructure, e.g., (1) small dataset size, in particular, a large amount of published data has not been used in building these models^30^, (2) model accuracy is currently not sufficient to make reliable predictions because of the use of inadequate algorithms, and (3) accurate interpretation of developed models for the inverse design of novel alloy materials is not straightforward. A well-integrated effort to alleviate each of these issues will help further advance this field for not only rupture life or rupture strength prediction in ferrous materials in high temperature power plants, but also similar properties in other classes of materials for various high impact scientific applications.

To address these concerns, a workflow combining ML with high quality experimental data has been developed to construct an accurate predictive model for rupture strength prediction in 9–12% Cr FMA and austenitic stainless steel. The workflow consists of the following steps:

1. Data preprocessing to convert the raw experimental data suitable for ML model training, e.g., removing features or instances encompassing missing values, imputation of the missing values using the mean of the rest of the values, and scaling the data using the mean and standard deviation of each feature.
2. Preliminary analysis of the data, e.g., correlation between different features and distribution of the data.
3. Training the ML algorithm using a fivefold cross validation scheme.
4. Identification of the importance of different features on the ML model parameters.

## Machine learning algorithms

In order to parameterize the functional relation between the rupture strength and physical and processing parameters, three algorithms were chosen, 1. Gaussian Process Regression (GPR), 2. Neural Network (NN), and 3. Gradient Boosted Decision Tree (GBDT). GPR is a nonparametric kernel based probabilistic regression model. It not only provides a prediction value but also provides a measurement of the aleatoric uncertainty of each prediction. GPR can adapt itself with the growing dataset size and will provide a measurement of uncertainty which is crucial for future design of experiment via Bayesian iterative active learning to minimize the experimental efforts required to exhaustively screen the whole alloy space of interest. However, the runtime of GPR grows as $\sim\;N^{3}$ with the dataset size, so it might become expensive as our dataset grows past a certain threshold. A GP is defined by the mean and covariance to represent the prediction and the uncertainty of prediction. Let $m\left( x \right)$ and $k(x, x^{\prime}|\theta )$ be the mean and covariance, respectively, then the GP can be represented as,

1 $$ m\left( x \right) = {\mathbb{E}}\left[ {f\left( x \right)} \right] $$

2 $$ k(x,x^{\prime}|\theta ) = {\mathbb{E}}\left[ {\left( {f\left( x \right) - m\left( x \right)} \right)\left( {f\left( {x^{\prime}} \right) - m\left( {x^{\prime}} \right)} \right)} \right] $$

3 $$ y\;or\;f\left( x \right) \sim {\mathcal{G}\mathcal{P}}(m\left( x \right), \;\;k(x, x^{\prime}|\theta )) $$

In this study, radial basis function kernel is used to parameterize the GPR. In the radial basis function, hyperparameter $\sigma_{f}$ and $\sigma_{l}$ (shown in Eq. 2) are optimized by minimizing the negative log likelihood function.

4 $$ k\left( {x, x^{\prime}|\theta } \right) = \sigma_{f}^{2} \exp \left( { - \frac{1}{2}\frac{{\left( {x_{i} - x_{j} } \right)^{T} \left( {x_{i} - x_{j} } \right)}}{{\sigma_{l}^{2} }}} \right) $$

Neural Networks (NN) have been developed to mimic the mathematical model representing the biological nervous system. The basic unit of an NN is a neuron or a node. Mathematically NN is composed of several layers of neurons, each connected to all the neurons in the preceding and succeeding layers except the input and output layer. The rationale for choosing NN is that the flexible nature of the architecture makes it a superior superset of all parametric regression models, as evidenced by the recent success of NN in several scientific and technological fields. However, the power of NN is only realized when the dataset volume is large enough to allow efficient learning of all the parameters of a complex system thus producing an accurate model. The network is trained via feedforward-backpropagation until the learning is complete which is characterized by the minimization of the loss function. In order to enforce non-linearity in the model, Rectified Linear Unit (ReLU), shown in Eq. (5), is used which scales the input linearly according to the learned weights and biases but clips the negative output to zero.

5 $$ f\left( x \right) = \left\{ {\begin{array}{*{20}l} {0,} \hfill & {x < 0} \hfill \\ {x,} \hfill & {x \ge 0} \hfill \\ \end{array} } \right. $$

Gradient Boosted Decision Tree (GBDT) is an ensemble of weak decision tree models. Unlike other common ensemble techniques, it iteratively fits the data and focuses on the points weakly described in the previous iteration. The principal idea is to build new base learners to be maximally correlated with the negative gradient of the loss function of the whole ensemble. Then the model estimates the function of future variable by the linear combination of the individual decision trees. For a medium sized scientific dataset (like the one used in this study), it is found to be more powerful than NN as the NN accuracy is constrained by the dataset volume.

## Results and discussion

**Figure 1:**
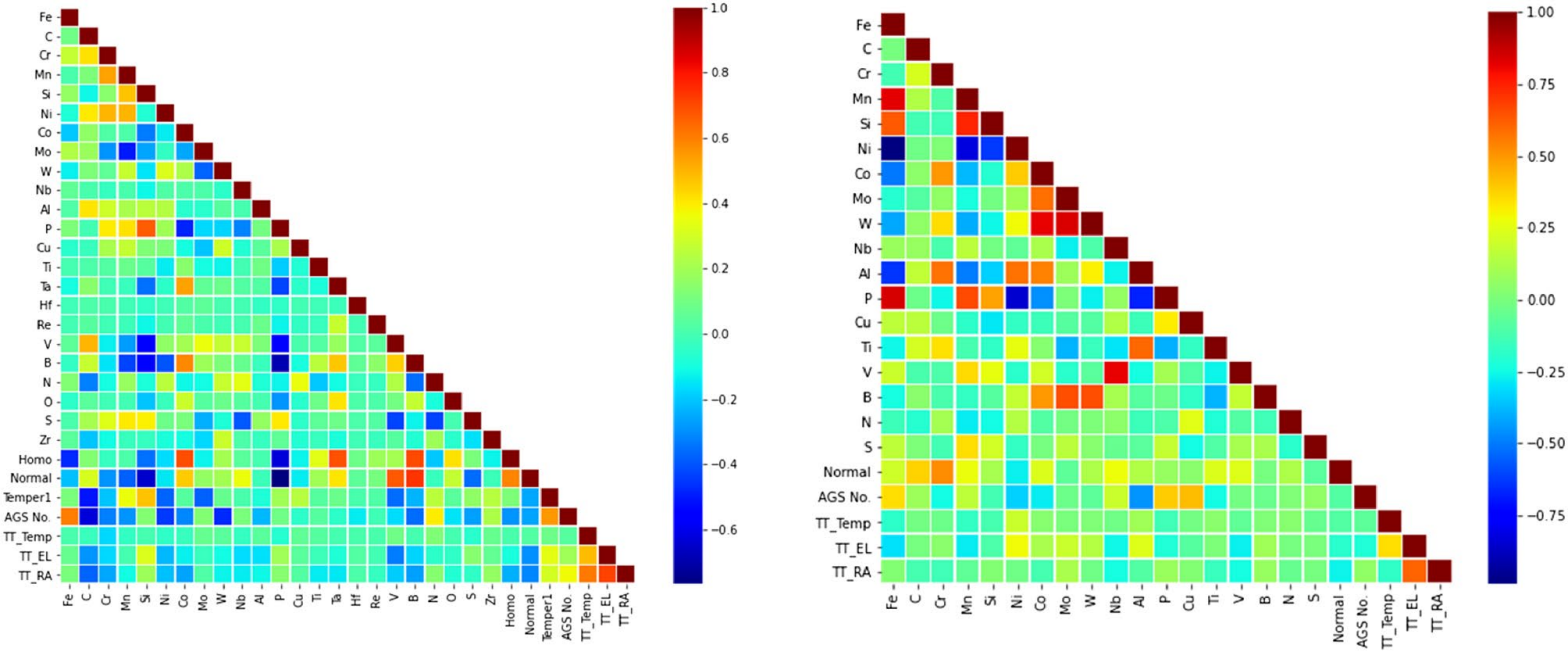
Pearson correlation coefficients of features for the 9–12% Cr FMA (left) and austenitic stainless steel (right).

The data used in this study were collected and compiled into a consistent and reliable set of data by the National Energy Technology Laboratory’s (NETL) effort on Extreme Environment Materials, eXtremeMAT (XMAT). XMAT is a collaborative undertaking between seven U.S. Department of Energy national laboratories with the goal of accelerating the development of improved heat resistant alloys for various components in fossil energy power plants and to predict the long-term performance of these alloys, e.g., rupture life and rupture strength. By utilizing state-of-the-art computational materials modeling and cutting-edge experimental tools across the DOE National Laboratories in conjunction with the industry partnership, XMAT is expected to accelerate the development and deployment of new heat resistant alloys for Fossil Energy applications. Two datasets, i.e., 9–12% Cr FMA and austenitic 347H stainless steel, are used to build the predictive models as both are very important for different components of a fossil energy power plant. The 9–12% Cr FMA dataset contains 1203 data instances with 30 features while the austenitic stainless steel dataset contains 823 data instances with 24 features after the preprocessing steps. In Table 1, various aspects of both datasets, including feature names, description, units, mean values, and standard deviations, etc., are tabulated. In Fig. 1, the Pearson correlation coefficient between the features is used to identify collinear features which may lead to fitting artifacts that are not easy to disentangle.

**Table 1:** Summary of the features present in both datasets, including feature name, description, units, mean values, and standard deviations.

Feature name	Description	Unit	9–12% Cr FMA		Austenitic stainless steel	
			Mean	Std.	Mean	Std.
Fe	Iron content	wt%	85.92	6.47	56.06	18.53
C	Carbon content	wt%	0.14	0.04	0.06	0.02
Cr	Chromium content	wt%	9.98	1.45	18.25	1.74
Mn	Manganese content	wt%	0.44	0.17	1.30	0.44
Si	Silicon content	wt%	0.25	0.16	0.52	0.14
Ni	Nickel content	wt%	0.28	0.23	21.76	18.41
Co	Cobalt content	wt%	0.72	1.44	0.14	0.26
Mo	Molybdenum content	wt%	0.84	0.52	1.09	1.88
W	Tungsten content	wt%	0.57	0.81	0.03	0.14
Nb	Niobium content	wt%	0.05	0.06	0.15	0.29
Al	Aluminum content	wt%	0.01	8.69 × 10^−3^	0.11	0.16
P	Phosphorous content	wt%	0.01	8.65 × 10^−3^	0.02	8.11 × 10^−3^
Cu	Copper content	wt%	0.06	0.16	0.21	0.49
Ti	Titanium content	wt%	4.4 × 10^−3^	0.02	0.16	0.19
Ta	Tantalum content	wt%	0.04	0.09		
Hf	Hafnium content	wt%	3.99 × 10^−3^	7.98 × 10^−3^		
Re	Rhenium content	wt%	1.33 × 10^−3^	0.01		
V	Vanadium content	wt%	0.17	0.09	4.24 × 10^−3^	0.01
B	Boron content	wt%	4.05 × 10^−3^	4.62 × 10^−3^	8.82 × 10^−4^	9.65 × 10^−4^
N	Nitrogen content	wt%	0.03	0.02	0.03	0.05
O	Oxygen content	wt%	1.93 × 10^−3^	6.89 × 10^−3^		
S	Sulfur content	wt%	4.75 × 10^−3^	3.84 × 10^−3^	9.00 × 10^−3^	5.79 × 10^−3^
Zr	Zirconium content	wt%	3.8 × 10 − 5	1.92 × 10^−4^		
Homo	Homogenization	Yes/no				
Normal	Normalization or austenization heat treatment temperature	℃	1075.46	77.77	1115.09	64.39
Temper1	Temper heat treatment 1	℃	685.49	75.61		
AGS no	Austenitic grain size number	$\frac{grains}{{{\text{mm}}^{2} }}$	6.49	2.34	4.76	1.55
TT_Temp	Test temperature	℃	369	229	477	269
TT_EL	Elongation to failure	%	25.61	13.04	52.31	18.21
TT_RA	Reduction in area	%	71.54	14.51	69.35	12.12

From the Pearson correlation coefficient analysis, no strong correlation was found amongst the features in the 9–12% Cr FMA dataset. However, some feature pairs in austenitic stainless steels dataset demonstrate strong correlation. Even though it is desirable to have low correlation amongst features present in the ML models, in this case it was considered a coincidence rather than correlation as those occurrences are only present for different chemical compositions.

To quantify the ML performance of different algorithms, the correlation coefficient ($R^{2}$) of a linear fit of the predicted data and the actual data is used. To avoid overfitting in the regression modeling and to collect sufficient statistics about the model performance on unseen data, fivefold cross validation was performed for each ML algorithm.

**Figure 2:**
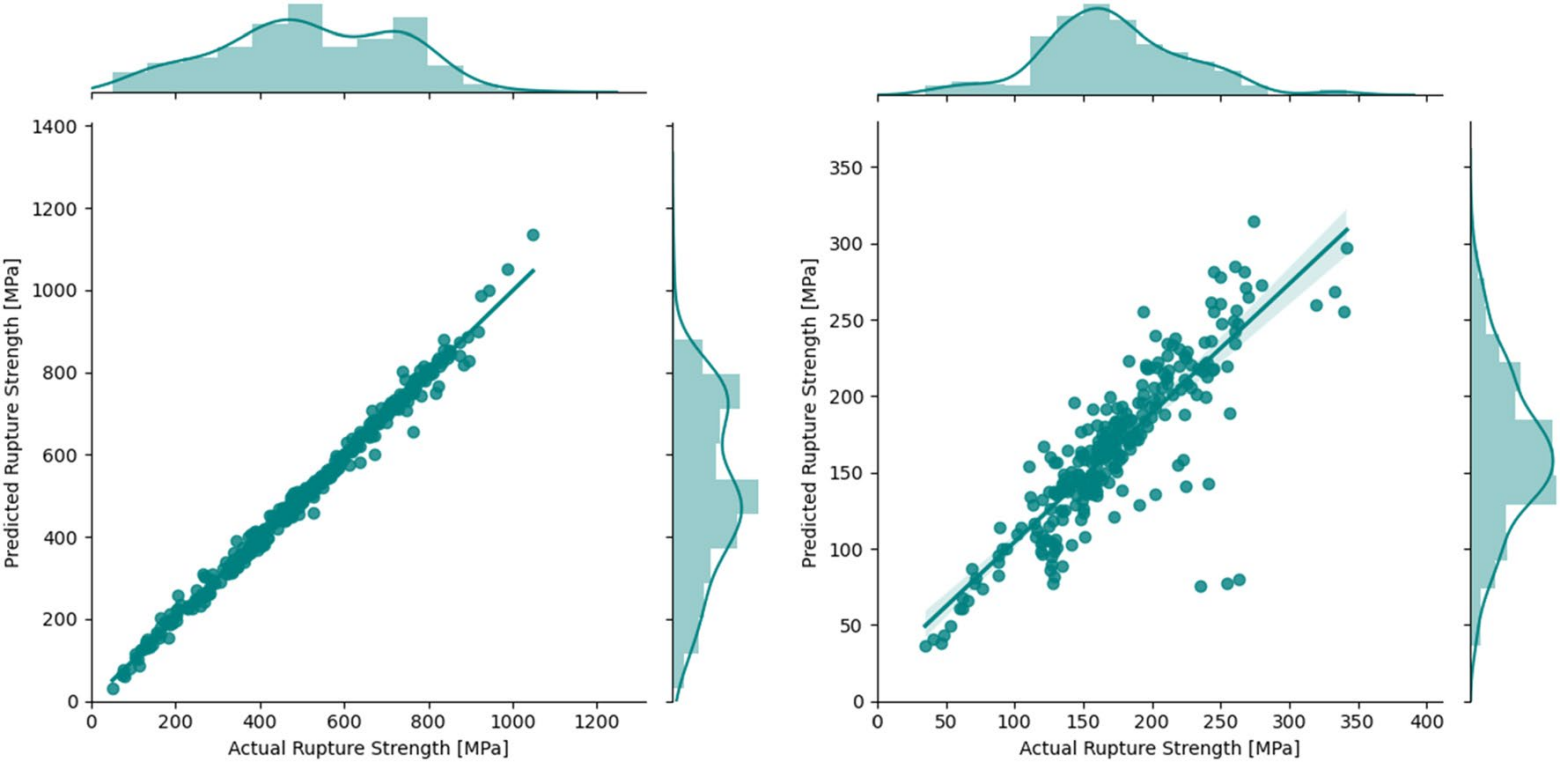
Parity plot for the testing data for GP regression for the 9–12% Cr FMA (left) and austenitic stainless steels (right).

First, a Gaussian Process (GP)^31^ regression with a combination of a radial basis function (RBF) and white kernel was used to train the model. The white kernel acts as a regularizer and accounts for noise by adding a constant to the diagonal elements of the covariance matrix. The *scikit-learn* Python package^32^ was used to train the model and *negative log likelihood* is used to optimize the kernel parameters. $R^{2}$ for the testing set are 0.92 and 0.83, respectively, for the 9–12% Cr FMA and austenitic stainless steels. In Fig. 2, the parity plots for the predicted strength and actual strength are shown for both datasets from the GP regression. The *R*^2^ value of the austenitic stainless steel model is less than that for the 9–12% Cr FMA model, mainly due to the dataset size as the latter is about one third smaller. The 9–12% Cr FMA dataset additionally has a more even distribution of rupture strength values across the possible range, where the austenitic stainless steel rupture strength values are more clustered around the center of the range. The austenitic stainless steel dataset is also missing some features that are present in the former.

In Table 2, both the training and testing set performance for GP, and all subsequent algorithms, are tabulated. From the table, it is evident that the model is not overfitting the data as the training and testing performance are quite similar. However, considering the performance of the state-of-the-art model ($R^{2}$ > 0.95) for yield stress prediction reported in the literature^33^ for a similar FMA dataset and the high variance of the $R^{2}$, this performance is deemed as unsatisfactory for accurate prediction of rupture strength. It is worth noting that in that study, in addition to the chemical composition and processing parameters, synthetic alloy features generated using CALPHAD were incorporated to capture microstructural and phase transformation related information.

**Table 2:** Summary of the ML model results in terms of correlation coefficient (*R*^2^) for both the training and hold out testing set.

	Gaussian process	Neural network	Gradient boosting machine
**9–12% Cr FMA**			
Training set	0.93 ± 0.05	0.94 ± 0.01	0.99 ± 2 × 10^−4^
Testing set	0.92 ± 0.07	0.93 ± 0.02	0.98 ± 4 × 10^−3^
**Austenitic stainless steel**			
Training set	0.91 ± 0.06	0.86 ± 0.02	0.99 ± 3 × 10^−4^
Testing set	0.83 ± 0.08	0.84 ± 0.02	0.95 ± 5 × 10^−3^

**Figure 3:**
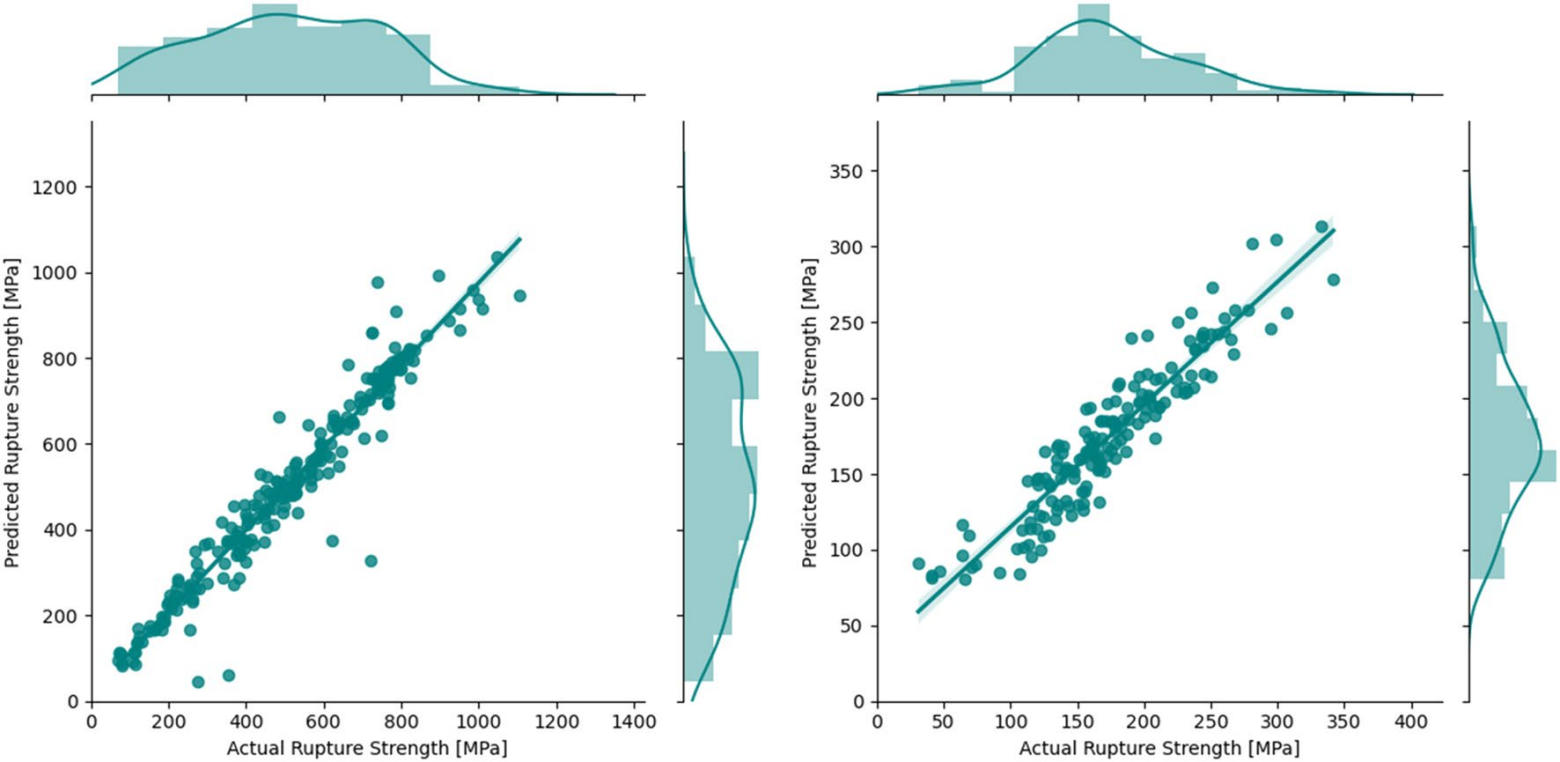
Parity plot for the testing data for NN regression for the 9–12% Cr FMA (left) and austenitic stainless steels (right).

Next, a Neural Network (NN) was trained to map the underlying correlations between the features and the target properties, i.e., rupture strength. The Keras Python package^34^ with TensorFlow backend^35^ was used to train the model. Two hidden layers, with 64 neurons each, were used in addition to the input layer and the output layer. It is worth noting that, a grid search optimization over the number of hidden layers and neurons in each layer was performed and based on the optimization two hidden layers with 64 neurons were chosen as the preferred architecture of the NN model. For the activation function, Rectified Linear Unit (ReLU) was used, and the models were trained for 4000 epochs to ensure convergence. The $R^{2}$ value for the testing sets are 0.93 and 0.84, respectively, for the 9–12% Cr FMA and the austenitic stainless steel datasets. In Fig. 3, ML-predicted rupture strength is plotted against the actual rupture strength. For the NN, similar performance was obtained as per the GP, though the variance of $R^{2}$ is smaller, indicating the model performance is robust. However, considering the success of Gradient Boosting Machine (GBM)^36^ in several scientific articles and ML competition, a GBM-based workflow was also built.

**Figure 4:**
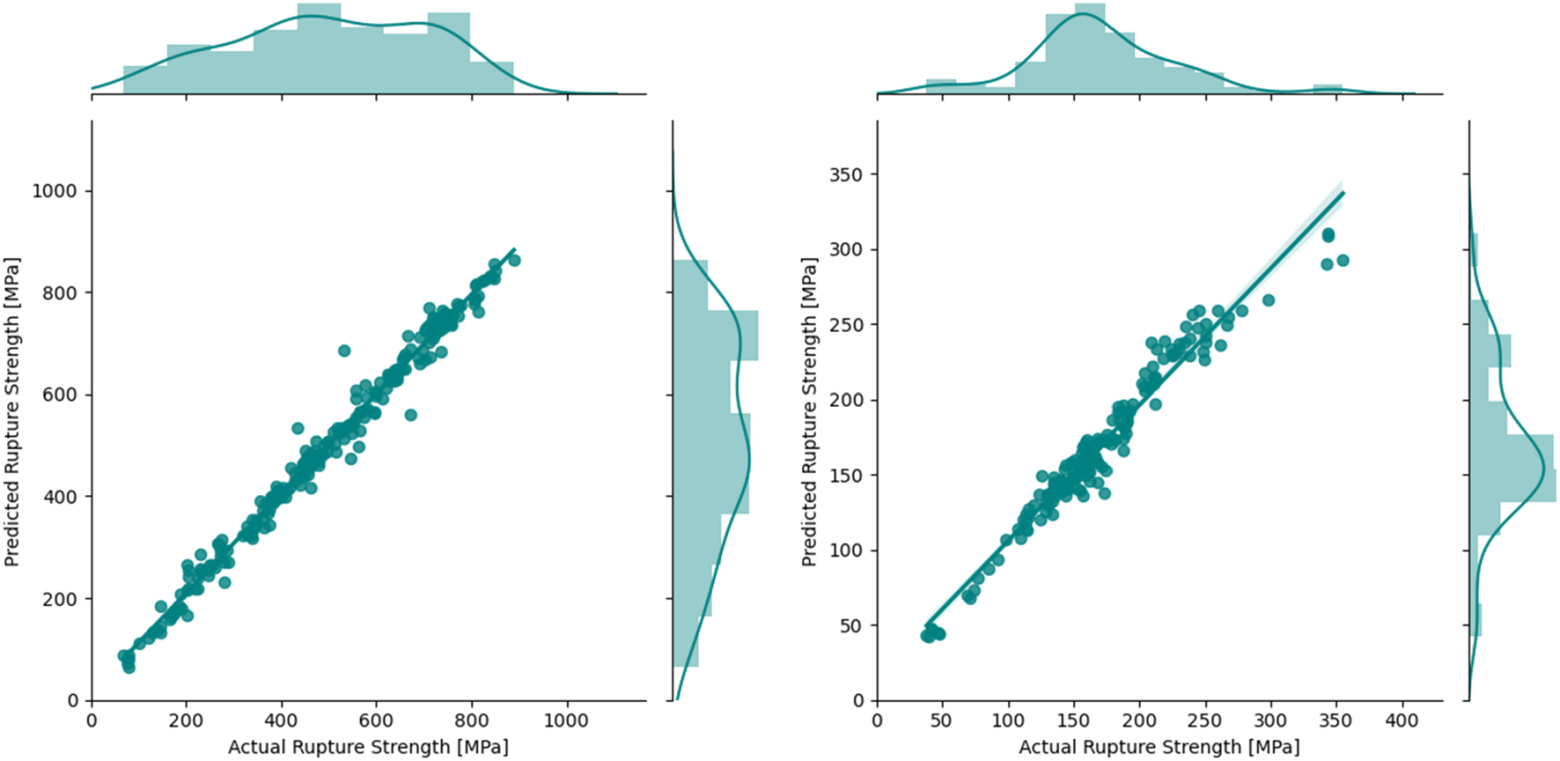
Parity plot for the testing data for GBDT regression for the 9–12% Cr FMA (left) and austenitic stainless steel (right).

As mentioned, a Gradient Boosted Decision Tree (GBDT) algorithm was trained to predict the rupture strength of 9–12% Cr FMA and austenitic stainless steel. To train the model, the *CatBoost* package with Python interface^37^ was used. In this case, $R^{2}$ values for the testing set are 0.98 and 0.95, respectively, for the 9–12% Cr FMA and the austenitic stainless steel datasets. In Fig. 4, the parity plots of the actual and predicted data are illustrated. Based on the performance of the three algorithms, the GBDT is the best algorithm for building a ML model conditioned on the chemical composition and processing related features for the accurate (mean of $R^{2}$ is high) and robust (variance of $R^{2}$ is low) prediction of rupture strength. Also, it was found that when the dataset volume is substantial, additional synthetic alloy features and/or an intermediate model for Prior Austenitic Grain Size (PAGS) are not needed to faithfully map the underlying functional relation between the features and the rupture strength.

**Figure 5:**
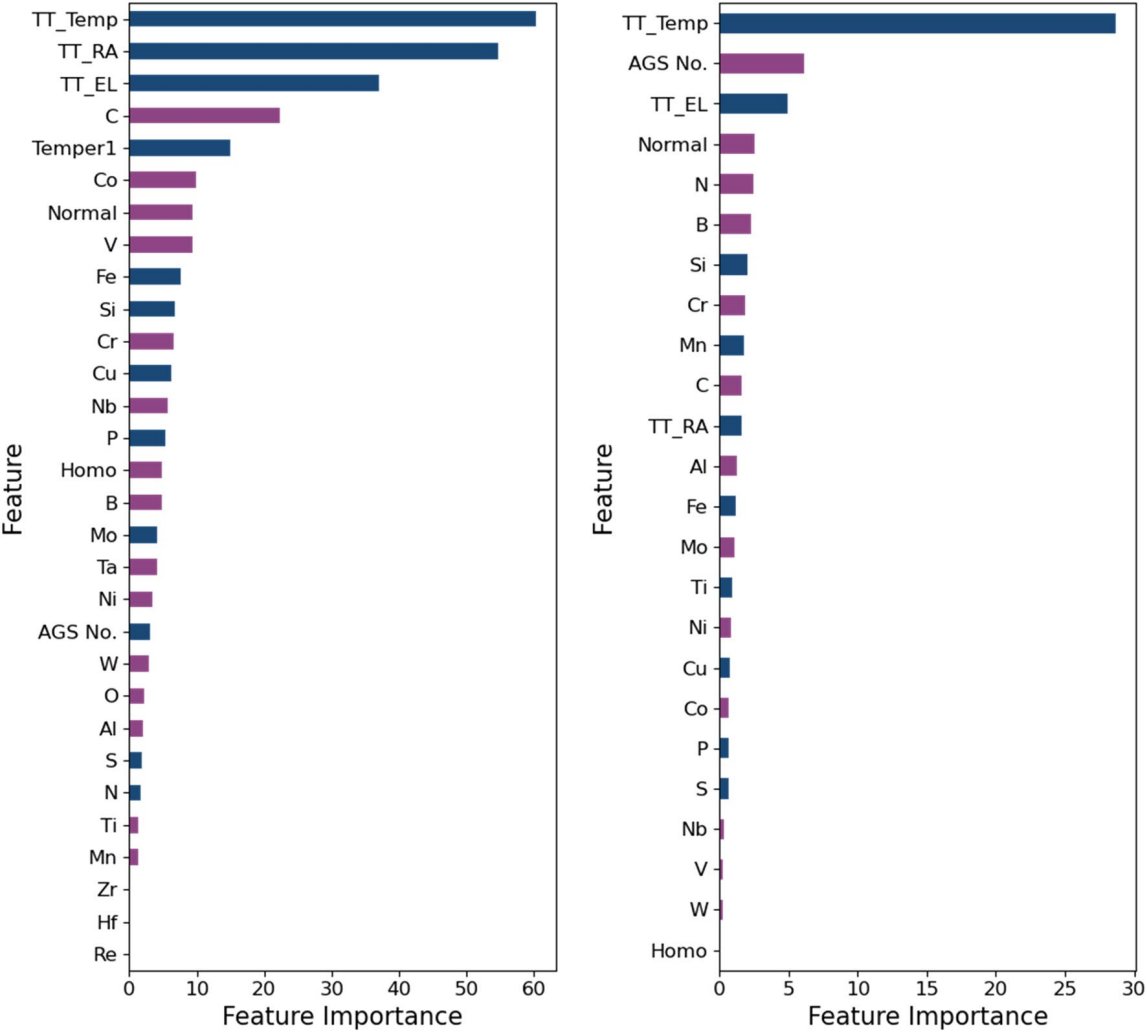
Importance of different features for the 9–12% Cr FMA (left) and austenitic stainless steel (right). The x-axis is plotted in terms of arbitrary unit to show the relative importance of different features, but the magnitude has no physical interpretation. Purple color indicates positive correlation while dark blue color indicates negative correlation.

At this point an investigation of the importance of various features on model performance was undertaken. Subsequently, the various features were investigated to quantify the effect of different features on model performance for the GBDT algorithm using the Shapley value^38,39^ analysis. In Fig. 5, the feature importance plot for both datasets is shown. For the 9–12% Cr FMA dataset, test temperature, reduction in area, and elongation to failure are the most important features. As for the chemical composition, carbon is the most important feature, as expected, with a positive correlation indicating having more carbon in the alloy increases the rupture strength of ferritic-martensitic steels. The negative correlation for the AGS No. indicates that to have higher rupture strength in 9–12% Cr FMA, it is essential to have a large austenitic grain size (small AGS number) to reduce the overall grain boundary line length, which is usually responsible for crack propagation^40^. However, AGS No. in 9–12% Cr FMA is not as important as austenitic steel as the strength of austenitic steel depends on its ability to trap carbon in the interstitial site of the austenitic grains which is not the case for 9–12% Cr FMA. For austenitic stainless steel, test temperature, austenitic grain size, and elongation to failure are the most important features. In contrast, the AGS No. is positively correlated to the rupture strength, meaning a larger grain size is detrimental to the rupture strength of the austenitic stainless steels. In the annealed condition, i.e., austenitic stainless steels, the YS increases with the decreasing grain size through an ordinary Petch-Hall relation^41^, which indicates the developed model is capable of differentiating the contrasting effect of grain size in 9–12% Cr FMA and austenitic steels. Another interesting observation is, microalloying elements, e.g., B, N, Si etc., are more important factors affecting the yield strength of austenitic 347H, which even surpasses the importance of reduction in area (2nd important feature in the 9–12% Cr FMA dataset). It is well known that addition of B, N, and other microalloying elements has a very perceptible positive impact on the growth of creep life in austenitic 347H^42^ which can explain why these microalloying elements are important features for the prediction of yield strength in austenitic 347H stainless steels.

It is of note that the information contained in the two datasets are for alloys that have been successfully developed, thoroughly tested and effectively deployed in the commercial power generation sector. Within that context certain trends noted previously must be qualified to a certain extent. For example, in the case of the 9–12% Cr FMA dataset, and with respect to chemical composition, the effect of carbon is well known. Increasing the carbon mass fraction creates strengthening carbides and saturates interstitial sites in the Fe matrix with C atoms. Both of these events strengthen the alloy. However, at some point carbon addition above this level becomes detrimental. This point falls outside the ranges of alloys studied since alloys with very high C contenat would have had properties that negated their further development. There are other examples where the effect of chemistry, and the trend influenced, are limited within a particular range. This example highlights the critical importance of having a thorough domain knowledge for the alloys being designed. Domain knowledge and physical laws are valuable to constrain data-driven models. Also, it is critical to include all data for alloy classes, not only the data for alloys that were deemed successful but also the data for those that were not.

## Conclusion

In summary, a workflow has been proposed and examined for 9–12% Cr FMA and austenitic stainless steel datasets for making efficient prediction of the rupture strength based on the GBDT algorithm. Based on the Shapley value analysis, important features were identified and the findings are rationalized in light of the domain knowledge. By integrating these ML models into the existing alloy design strategy, significant acceleration may be gained for the identification of promising 9–12% Cr FMA or austenitic stainless steels with superior creep rupture strength properties.

## Data Availability

The data used that support the findings of this study are available upon request to J.H (Jeffrey.Hawk@NETL.DOE.GOV).

## References

[1] Chen XH, Lu J, Lu L, Lu K (2005). Tensile properties of a nanocrystalline 316L austenitic stainless steel. Scr. Mater..

[2] Sourmail T (2001). Precipitation in creep resistant austenitic stainless steels. Mater. Sci. Technol..

[3] Yamamoto Y (2008). Alloying effects on creep and oxidation resistance of austenitic stainless steel alloys employing intermetallic precipitates. Intermetallics.

[4] Yamamoto Y (2007). Creep-resistant, Al_2_O_3_-forming austenitic stainless steels. Science (80-).

[5] Bengochea R, Lopez B, Gutierrez I (1998). Microstructural evolution during the austenite-to-ferrite transformation from deformed austenite. Metall. Mater. Trans. A.

[6] Militzer M, Mecozzi MG, Sietsma J, Van der Zwaag S (2006). Three-dimensional phase field modelling of the austenite-to-ferrite transformation. Acta Mater..

[7] Klueh RL, Nelson AT (2007). Ferritic/martensitic steels for next-generation reactors. J. Nucl. Mater..

[8] Klueh RL (2002). Ferritic/martensitic steels—overview of recent results. J. Nucl. Mater..

[9] Bischoff J (2013). Corrosion of ferritic–martensitic steels in steam and supercritical water. J. Nucl. Mater..

[10] Kapoor M (2014). Aging characteristics and mechanical properties of 1600 MPa body-centered cubic Cu and B2-NiAl precipitation-strengthened ferritic steel. Acta Mater..

[11] Tian J (2018). Role of Co in formation of Ni–Ti clusters in maraging stainless steel. J. Mater. Sci. Technol..

[12] Leitner H, Schober M, Schnitzer R, Zinner S (2011). Strengthening behavior of Fe–Cr–Ni–Al–(Ti) maraging steels. Mater. Sci. Eng. A.

[13] Ågren J (1996). Calculation of phase diagrams: Calphad. Curr. Opin. Solid State Mater. Sci..

[14] Vasudevan M, Venkadesan S, Sivaprasad PV, Mannan SL (1994). Use of the Larson–Miller parameter to study the influence of ageing on the hardness of cold-worked austenitic stainless steel. J. Nucl. Mater..

[15] Hohenberg P, Kohn W (1964). Inhomogeneous electron gas. Phys. Rev..

[16] Kohn W, Sham LJ (1965). Self-consistent equations including exchange and correlation effects. Phys. Rev..

[17] Rahman A (1964). Correlations in the motion of atoms in liquid argon. Phys. Rev..

[18] Alder BJ, Wainwright TE (1959). Studies in molecular dynamics. I. General method. J. Chem. Phys..

[19] Chen L-Q (2002). Phase-field models for microstructure evolution. Annu. Rev. Mater. Res..

[20] Boettinger WJ, Warren JA, Beckermann C, Karma A (2002). Phase-field simulation of solidification. Annu. Rev. Mater. Res..

[21] Steinbach I (2009). Phase-field models in materials science. Model. Simul. Mater. Sci. Eng..

[22] Ennis PJ, Zielinska-Lipiec A, Wachter O, Czyrska-Filemonowicz A (1997). Microstructural stability and creep rupture strength of the martensitic steel P92 for advanced power plant. Acta Mater..

[23] Butler KT, Davies DW, Cartwright H, Isayev O, Walsh A (2018). Machine learning for molecular and materials science. Nature.

[24] de Pablo JJ (2019). New frontiers for the materials genome initiative. NPJ Comput. Mater..

[25] Mamun O, Winther KT, Boes JR, Bligaard T (2020). A Bayesian framework for adsorption energy prediction on bimetallic alloy catalysts. NPJ Comput. Mater..

[26] Chatzidakis S, Alamaniotis M, Tsoukalas LH (2014). Creep rupture forecasting: a machine learning approach to useful life estimation. Int. J. Monit. Surveill. Technol. Res..

[27] Shin D, Yamamoto Y, Brady MP, Lee S, Haynes JA (2019). Modern data analytics approach to predict creep of high-temperature alloys. Acta Mater..

[28] Jiang X (2020). A strategy combining machine learning and multiscale calculation to predict tensile strength for pearlitic steel wires with industrial data. Scr. Mater..

[29] Liu Y (2020). Predicting creep rupture life of Ni-based single crystal superalloys using divide-and-conquer approach based machine learning. Acta Mater..

[30] Panchal JH, Kalidindi SR, McDowell DL (2013). Key computational modeling issues in integrated computational materials engineering. CAD Comput. Aided Des..

[31] Williams, C. K. I. & Rasmussen, C. E. Gaussian processes for regression. In *Advances in Neural Information Processing Systems* 514–520 (1996).

[32] Pedregosa F (2011). Scikit-learn: machine learning in Python. J. Mach. Learn. Res..

[33] Peng J, Yamamoto Y, Hawk JA, Lara-Curzio E, Shin D (2020). Coupling physics in machine learning to predict properties of high-temperatures alloys. NPJ Comput. Mater..

[34] François, C. Keras: The Python deep learning library. Accessed Aug 2020. https://keras.io (2015).

[35] Abadi, M. *et al.* Tensorflow: a system for large-scale machine learning. In *12th {USENIX} Symposium on Operating Systems Design and Implementation ({OSDI} 16)* 265–283 (2016).

[36] Song K, Yan F, Ding T, Gao L, Lu S (2020). A steel property optimization model based on the XGBoost algorithm and improved PSO. Comput. Mater. Sci..

[37] Prokhorenkova, L., Gusev, G., Vorobev, A., Dorogush, A. V. & Gulin, A. CatBoost: unbiased boosting with categorical features. In *Advances in Neural Information Processing Systems* 6638–6648 (2018).

[38] Winter E (2002). The Shapley value. Handb. Game Theory Econ. Appl..

[39] Lundberg, S. M. & Lee, S.-I. A unified approach to interpreting model predictions. In *Advances in Neural Information Processing Systems* 4765–4774 (2017).

[40] Yanagimoto F (2019). Contribution of grain size to resistance against cleavage crack propagation in ferritic steel. Acta Mater..

[41] Norström L-Å (1977). The influence of nitrogen and grain size on yield strength in Type AISI 316L austenitic stainless steel. Met. Sci..

[42] Xu Y (2010). Growth of creep life of type-347H austenitic stainless steel by micro-alloying elements. Mater. Sci. Eng. A.

